# Dengue Virus Exported from Côte d’Ivoire to Japan, June 2017

**DOI:** 10.3201/eid2310.171132

**Published:** 2017-10

**Authors:** Tetsuya Suzuki, Satoshi Kutsuna, Satoshi Taniguchi, Shigeru Tajima, Takahiro Maeki, Fumihiro Kato, Chang-Kweng Lim, Masayuki Saijo, Motoyuki Tsuboi, Kei Yamamoto, Shinichiro Morioka, Masahiro Ishikane, Kayoko Hayakawa, Yasuyuki Kato, Norio Ohmagari

**Affiliations:** National Center for Global Health and Medicine, Tokyo, Japan (T. Suzuki, S. Kutsuna, M. Tsuboi, K. Yamamoto, S. Morioka, M. Ishikane, K. Hayakawa, Y. Kato, N. Ohmagari);; National Institute of Infectious Diseases, Tokyo (S. Taniguchi, S. Tajima, T. Maeki, F. Kato, C.-K. Lim, M. Saijo);; Tohoku University, Sendai, Japan (M. Ishikane)

**Keywords:** dengue fever, Africa, Abidjan, Côte d’Ivoire, Japan, outbreak, viruses, vector-borne infections

## Abstract

Since April 2017, a dengue fever outbreak has been ongoing in Côte d’Ivoire. We diagnosed dengue fever (type 2 virus) in a traveler returning to Japan from Côte d’Ivoire. Phylogenetic analysis revealed strain homology with the Burkina Faso 2016 strain. This case may serve as an alert to possible disease spread outside Africa.

In recent decades, dengue virus (DENV) infection has been spreading worldwide. Although in Africa the leading cause of acute febrile illness is still malaria, dengue has recently gained momentum (*1*). Dengue has been reported in 34 African countries, although it has probably been underreported because of the lack of diagnostic testing and systematic surveillance in Africa (*2*). Four types of virus have been isolated; the most endemic to Africa is DENV type 2 (DENV-2), followed by DENV-1 (*2*). The first reported case of DENV-1 infection occurred in a young soldier from Abidjan, Côte d’Ivoire, in 1999 (*3*). At that time, no other similar cases or epidemics in Abidjan had been reported. In 2008, a closely related strain, DENV-3, was isolated from visitors to Côte d’Ivoire (*4*,*5*). In 2010, dengue fever was biologically confirmed for 7 patients who had never been in a dengue-endemic area, and DENV-3 was confirmed by reverse transcription PCR for 4 of these patients (*6*). A prospective study in Abidjan also revealed that DENV-3 had been the cause of febrile illness during 2011–2012 (*7*). Thus, DENV-3 may have circulated widely in Côte d’Ivoire, especially in Abidjan. During the 2016 outbreak in Burkina Faso, DENV-2 infection was detected in 2 travelers returning from Burkina Faso to France (*8*). During August–November 2016, the World Health Organization reported 1,061 probable dengue cases and 15 deaths from dengue (*9*). We report a case of dengue fever exported to Japan from Abidjan in 2017.

On June 19, 2017, a man in his early 50s sought care at the Center Hospital of the National Center for Global Health and Medicine, Tokyo, Japan, for fever, chills, headache, and mild joint pain. In June 2013, he had traveled to Abidjan for business, and on June 13, 2017, he returned to Japan. He had been vaccinated for yellow fever. He had noticed a high fever in the morning and sought care the same evening. 

Physical examination revealed body temperature of 39.3°C, mildly hyperemic conjunctiva, and a slight rash on his trunk. His blood biochemistry profile showed 3,640 × 10^9^ leukocytes/L, hemoglobin level 13.5 g/dL, and 151 × 10^9^ thrombocytes/L. Results of a rapid diagnostic test for malaria (BinaxNOW Malaria; Alere, Waltham, MA, USA) were negative. A thin-coated peripheral blood smear with May-Grünwald Giemsa stain showed no *Plasmodium* parasites. Results of a dengue rapid diagnostic test (Dengue Duo NS1 Ag + Ab Combo; Alere) were negative for IgM and IgG but positive for nonstructural protein 1 antigen. Serum samples obtained on June 19 and 26 were sent for real-time reverse transcription PCR to the National Institute of Infectious Diseases, Tokyo, where DENV-2 RNA was detected. 

The patient’s signs and symptoms resolved spontaneously in a week; his lowest thrombocyte count was 99 × 10^9^ thrombocytes/L. On June 19, a diagnostic test for DENV IgM (Dengue Virus IgM Capture ELISA; Focus Diagnostics, Cypress, CA, USA) yielded negative results; however, on June 26, positive results indicated seroconversion. 

Phylogenetic analysis of the DENV envelope gene indicated that the sequence of DENV-2 obtained from the patient belonged to the cosmopolitan genotype and was 99% identical with the envelope gene of DENV-2 strains from the 2016 dengue epidemic in Burkina Faso (GenBank accession nos. LC206003, KY627763, and KY627762) (Figure). The sequence of DENV-2 from the patient also showed 97% identity with that of the DENV-2 strains from the 1983 (accession no. EU056810) and 1986 (accession nos. HM234642 and GU131843) epidemics in Burkina Faso. Strains of DENV-2 from the 2005 epidemic in Ghana (accession no. EU005258) shared 95% identity with that of the patient reported here.

**Figure:**
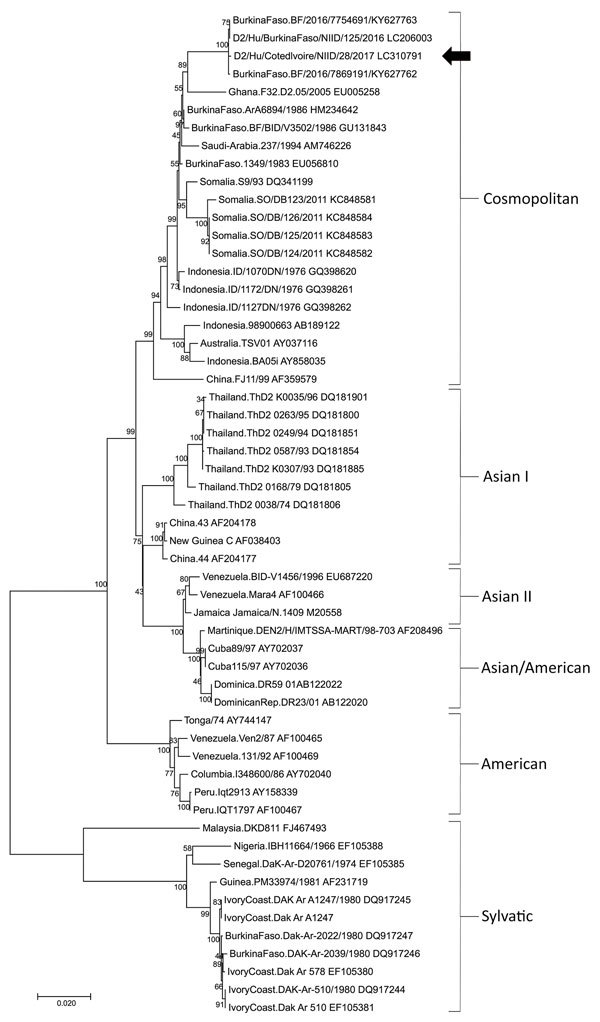
Comparison of dengue virus type 2 from a patient returning from Abidjan, Côte d’Ivoire, to Japan (arrow) with reference dengue virus sequences. Virus lineages are shown on right. Phylogenetic trees were constructed by using the neighbor-joining method. The maximum composite likelihood method was used, and rates among sites were uniform. Analyses were performed by using MEGA6 software (http://megasoftware.net). Scale bar indicates substitutions per nucleotide position.

Phylogenetic analysis indicated that the dengue virus genome sequence in this case is highly homologous with recent strains in Africa, especially from the 2016, 1986, and 1983 outbreaks in Burkina Faso. Similar strains of DENV-2 have repeatedly caused outbreaks in West Africa (*8*). As of April 2017, DENV-2 and DENV-3 have been isolated from patients in the ongoing outbreak in Abidjan (*10*). Although no cases have been reported outside Abidjan, the case reported here may be a sentinel case, serving as an alert to the possibility of disease spread outside Africa.

## References

[1] Freedman DO, Weld LH, Kozarsky PE, Fisk T, Robins R, von Sonnenburg F, et al.; GeoSentinel Surveillance Network. Spectrum of disease and relation to place of exposure among ill returned travelers. N Engl J Med. 2006;354:119–30. 10.1056/NEJMoa051331 16407507

[2] Amarasinghe A, Kuritsk JN, Letson GW, Margolis HS. Dengue virus infection in Africa. Emerg Infect Dis. 2011;17:1349–54. 21801609 10.3201/eid1708.101515 PMC3381573

[3] Durand JP, Vallée L, de Pina JJ, Tolou H. Isolation of a dengue type 1 virus from a soldier in West Africa (Côte d’Ivoire). Emerg Infect Dis. 2000;6:83–4. 10.3201/eid0601.000116 10653577 PMC2627986

[4] Ninove L, Parola P, Baronti C, De Lamballerie X, Gautret P, Doudier B, et al. Dengue virus type 3 infection in traveler returning from west Africa. Emerg Infect Dis. 2009;15:1871–2. 10.3201/eid1511.081736 19891895 PMC2857216

[5] Moi ML, Takasaki T, Kotaki A, Tajima S, Lim CK, Sakamoto M, et al. Importation of dengue virus type 3 to Japan from Tanzania and Cote d’Ivoire. Emerg Infect Dis. 2010;16:1770–2. 10.3201/eid1611.101061 21029541 PMC3294538

[6] Aoussi EB, Ehui E, Kassi NA, Kouakou G, Nouhou Y, Adjogoua EV, et al. Seven native cases of dengue in Abidjan, Ivory Coast. Med Mal Infect. 2014;44:433–6. 10.1016/j.medmal.2014.08.002 25239146

[7] L’Azou M, Succo T, Kamagaté M, Ouattara A, Gilbernair E, Adjogoua E, et al. Dengue: etiology of acute febrile illness in Abidjan, Côte d’Ivoire, in 2011-2012. Trans R Soc Trop Med Hyg. 2015;109:717–22. 10.1093/trstmh/trv076 26385938 PMC4603269

[8] Eldin C, Gautret P, Nougairede A, Sentis M, Ninove L, Saidani N, et al. Identification of dengue type 2 virus in febrile travellers returning from Burkina Faso to France, related to an ongoing outbreak, October to November 2016. Euro Surveill. 2016;21:30425. 10.2807/1560-7917.ES.2016.21.50.30425 28006651 PMC5291134

[9] World Health Organization. Dengue fever–Burkina Faso. Disease Outbreak News, 18 November 2016 [cited 2017 Jul 13]. http://www.who.int/csr/don/18-november-2016-dengue-burkina-faso/en/.

[10] World Health Organization, Regional Office for Africa, Health Emergencies Programme. Outbreaks and other emergencies updates; week 22: May 27–June 2, 2017 [cited 2017 Jun 24]. http://www.afro.who.int/health-topics/disease-outbreaks/outbreaks-and-other-emergencies-updates

